# Impact of age-related gut microbiota dysbiosis and reduced short-chain fatty acids on the autonomic nervous system and atrial fibrillation in rats

**DOI:** 10.3389/fcvm.2024.1394929

**Published:** 2024-06-12

**Authors:** Li Liu, Yingqi Yi, Rong Yan, Rong Hu, Weihong Sun, Wei Zhou, Haiyan Zhou, Xiaoyun Si, Yun Ye, Wei Li, Jingjing Chen

**Affiliations:** ^1^ Department of Cardiovascular Medicine, Affiliated Hospital of Guizhou Medical University, Guiyang, China; ^2^ Translational Medicine Research Center, Guizhou Medical University, Guiyang, China; ^3^ Department of Cardiovascular Medicine, Guizhou Provincial People’s Hospital, Guiyang, China

**Keywords:** atrial fibrillation, gut microbiota, short-chain fatty acids, autonomic nervous system, aging

## Abstract

**Objective:**

Aging is the most significant contributor to the increasing prevalence of atrial fibrillation (AF). Dysbiosis of gut microbiota has been implicated in age-related diseases, but its role in AF development remains unclear. This study aimed to investigate the correlations between changes in the autonomic nervous system, short-chain fatty acids (SCFAs), and alterations in gut microbiota in aged rats with AF.

**Methods:**

Electrophysiological experiments were conducted to assess AF induction rates and heart rate variability in rats. 16S rRNA gene sequences extracted from fecal samples were used to assess the gut microbial composition. Gas and liquid chromatography–mass spectroscopy was used to identify SCFAs in fecal samples.

**Results:**

The study found that aged rats exhibited a higher incidence of AF and reduced heart rate variability compared to young rats. Omics research revealed disrupted gut microbiota in aged rats, specifically a decreased Firmicutes to Bacteroidetes ratio. Additionally, fecal SCFA levels were significantly lower in aged rats. Importantly, correlation analysis indicated a significant association between decreased SCFAs and declining heart rate variability in aged rats.

**Conclusions:**

These findings suggest that SCFAs, as metabolites of gut microbiota, may play a regulatory role in autonomic nervous function and potentially influence the onset and progression of AF in aged rats. These results provide novel insights into the involvement of SCFAs and autonomic nervous system function in the pathogenesis of AF. These results provide novel insights into the involvement of SCFAs and autonomic nervous system function in the pathogenesis of AF.

## Introduction

Atrial fibrillation (AF) is the most common cardiac arrhythmia in clinical practice, caused by multiple risk factors and resulting in significant morbidity and mortality (1, 2). Advanced age is an important and independent risk factor for AF, with a reported prevalence of 0.12%–0.16% in those younger than 50 years and 20% in those aged 80 years or older (3). The higher prevalence of AF in older adults can be attributed to degenerative changes in the aging heart, as well as the presence of comorbidities such as coronary artery disease, heart failure, and hypertension (4). Moreover, the recurrence rate of AF remains high in aged patients due to the complexity of its underlying mechanism and the diversity of complications (5) Although the exact mechanism underlying the relationship between aging and AF is not fully understood, research suggests that aging affects the structure and function of the autonomic nervous system (ANS), leading to changes in sympathetic and vague nerve activity and neurotransmitter release in the atrium, which play a significant role in the occurrence of AF (6, 7) Therefore, understanding the relationship between the ANS and AF in the elderly is crucial for the prevention and treatment of AF in this population. Currently, treatments for AF in older adults are limited. Traditional antiarrhythmic and anticoagulant drug treatments have limitations and risks in elderly patients. Thus, there is an urgent need for a better understanding of the disease and the discovery of novel therapeutic strategies to promote earlier treatment and prevention of age-related AF.

The gut microbiota refers to the diverse community of microorganisms, including bacteria, fungi, viruses, archaea, and protozoa, that reside in the human intestine. These microorganisms have a significant impact on both health and disease (8). Recently, there has been growing interest in the role of gut microbiota in the development of cardiovascular disease (CVD). Several studies have confirmed a correlation between changes in the composition of gut microbiota and the pathogenesis of CVD (9). These studies have demonstrated the existence of a “gut-brain-heart axis” (10), with disturbance of the gut microbiota considered as one of the potential mechanisms of various CVD (9, 11). Additionally, studies have shown that disturbance of gut microbiota may be related to the occurrence and development of AF in the elderly (12). Interestingly, disruption of the gut microbiota leads to reduced production of SCFAs (13), which are thought to have regulatory effects on the ANS (14). Therefore, we hypothesize that the disruption of the gut microbiota and decreased production of SCFAs during the aging process might be linked to ANS dysfunction and could significantly contribute to the onset and progression of AF among elderly individuals. However, there is currently a lack of sufficient evidence to support the correlation between SCFAs and AF, age-related gut microbiota disorders, and ANS dysfunction in the elderly.

The aim of this study is to investigate the relationship between gut microbiota disorder, SCFAs, and ANS dysfunction in aged rats with AF. Additionally, it aims to explore the impact of gut microbiota disorder on SCFAs and ANS dysfunction in aged rats. The study also examines the potential consequences of reduced production of SCFAs and its contribution to the development and progression of AF in aged rats. To achieve these objectives, we will utilize various strategies and methods including animal model experiments, 16S rRNA gene sequencing, targeted metabolomics, Langendorff *in vitro* perfusion, isolated cardiac electrophysiological examination, electrical mapping, and other technologies. Our objective is to comprehensively investigate the correlation between gut microbiota disorder, SCFAs, and ANS dysfunction in aged rats with AF, establishing a novel scientific foundation for the prevention and treatment of AF in the elderly. In summary, this study will enhance our understanding of the mechanism and development process of AF in the elderly and provide new ideas and strategies for the intervention and management of AF in this population.

## Materials and methods

### Animal preparation

Twelve male Sprague-Dawley (SD) rats of SPF grade were used in this study. Six young rats were aged 2–3 months with a body weight of 290–310 g, while six aging rats were aged 20–22 months with a body weight of 790–810 g. All rats were purchased from Chengdu Dasuo Laboratory Animal Technology Co, Ltd [Certificate No.SCXK (II) 2020-0030, China]. The rats were housed in the SPF-class animal room at the Animal Experiment Center of Guizhou Medical University. The room maintained a temperature of 20–25 °C, humidity of 50%–60%, and a 12-h light/dark cycle. The rats were allowed free access to water and food during a 7-day adaptive feeding period prior to the experiment. All animal experiments were conducted in accordance with the guidelines provided by the NIH Guide for the Care and Use of Laboratory Animals and were approved by the Animal Experiments Ethical Committee of Guizhou Medical University (Certificate No. 2304563).

### Left atrial diameter size, HRV, and *ex vivo* cardiac electrophysiological study

Echocardiography was conducted to evaluate the size of the left atrial diameter. To investigate whether aging is associated with increased susceptibility to atrial AF in rats, an atrial electrophysiological test was performed on young and aged rats.

Rats were anesthetized by intraperitoneal injection of 3% sodium pentobarbital (60 mg/kg). After reaching a state of deep anesthesia, they were promptly placed on a temperature-controlled operating table. The Power Lab data acquisition system (AD Instruments, Colorado Springs, CO) was used to record rat electrocardiograms. ECGs that were free of arrhythmia, interference, and artifacts for a duration of at least 2 min were selected for heart rate variability analysis, which was performed using EMap Record 5.0 software (Mapping Lab Ltd., UK). The heart rate variability (HRV) analysis parameter, SDRR, PR, RR, was determined.

Langendorff-perfused rat hearts were prepared using the methodology described in a previous study (15). Anesthetized rats were injected with heparin (3,125 U/kg) for 15 min. Subsequently, the rats were euthanized by cervical dislocation. Open thoracotomy was immediately performed to remove the heart, which was then promptly placed in a Krebs-Henseleit (KH) solution. The KH solution consisted of the following components: NaCl 120 mM, KCl 4.5 mM, CaCl2 1.25 mM, MgCl2 6H2O 1.2 mM, KH2PO4 1.2 mM, NaHCO3 20 mM, C6H12O6 10 mM, with a pH of 7.4. The aortas were cannulated, and the hearts were perfused using a non-recirculating system with a Langendorff apparatus (Harvard Bioscience Co., Ltd, Germany). The perfusion solution, consisting of a K-H solution bubbled with 95% O2 and 5% CO2, was delivered at a constant pressure of 70 mmHg from oxygenated glass reservoirs at a temperature of (37 ± 0.5) °C. Perfusion was initiated within 2 min of heart removal. After a stabilization period of 20 min, rats with heart rates below 180 bpm were excluded from the study.

To induce AF, the following steps were taken: electrocardiogram (ECG) electrodes were attached to the sinus node and the apex of the heart. Stimulation electrodes were attached to the right atrial appendage, and matrix multi-channel electrophysiological mapping pen electrodes (PA06408080301) were attached to the left atrium (LA), displaying channels 1–64. A burst stimulation of 50 Hz lasting 2 s was continuously delivered for a total of 12 bursts. AF lasting more than 2 s was considered inducible AF. Subsequently, a 64-channel MEA mapping system (EMS64-USB-1003, Mapping Lab Ltd., UK) was used to collect data for measuring conduction velocity and dispersion. The collected cardiac electrical conduction data were analyzed using EMap Record 5.0 software (Mapping Lab Ltd., UK) and further analyzed with Emap Scope 5.0 software (Mapping Lab Ltd., UK). Isochrones were extracted from these recordings using the program's built-in functions, and atrial conduction velocities were calculated for 64 channels over 5 cardiac cycles. The P5, P50, and P95 data were obtained based on the histogram of the local maximum phase difference. The values P95-P5 and P95-P5/P50 were normalized to 1 mm, and the absolute inhomogeneity (P95-P5) and inhomogeneity index (P95-P5/P50) of conduction were calculated.

## 16S rRNA gene sequencing

Fecal samples were collected from rats and immediately frozen at −80 °C for analysis. The samples were pulverized using a mortar and pestle, and the bacterial genomic DNA was extracted using the standard Power Soil kit protocol. Thawed fecal samples were mixed with MoBio lysis buffer and vortexed. The resulting fecal suspensions were centrifuged, and the supernatant was transferred to MoBio Garnet bead tubes containing MoBio buffer. The V3-V5 regions of the 16s rRNA gene sequences were extracted from the fecal samples using the Roche 454 sequencing system. Using diluted genomic DNA as a template, the V4 region of the bacterial 16S rRNA was amplified by PCR with the specific primers 515F(5′-GTGCCAGCMGCCGCGGTAA-3′) and 806R(5′-GGACTACHVGGGTWTCTAAT-3′) labeled in a 12 bp barcode. The gene sequences were then PCR amplified with barcoded universal primers. Sequences with a length of less than 200 bp or greater than 1,000 bp, as well as sequences with barcode mismatches, ambiguous bases, homopolymer runs exceeding six bases, and primer mismatches, were excluded. The remaining sequences were assigned to operational taxonomic units (OTUs) based on >97% pairwise sequence identity. Taxonomic classification of the OTUs was performed using the Ribosomal Database Project (RDP) reference database.

Expanded materials and methods are available in the Supplementary Material.

### Short-chain fatty acids (SCFAs) identification

First, sample preparation was conducted as follows: (1) 50 mg of fecal samples were pre-cooled at 4 °C and then added to a 1 M NaOH solution (5 mmol/L). (2) The mixture was homogenized for 3 min using a pre-cooled sample tray at −20 degrees and then ultrasonically extracted for 7 min in an ice bath. (3) After centrifugation at 12,000 r.p.m. for 10 min at 4 °C, 500 μl of supernatant was transferred into an injection vial, and 300 μl of pure water was added.

Next, sample derivation was conducted as follows: (1) Into the injection vial, add 500 μl of propanol/pyridine (3:2, v/v) and 100 μl of propyl chloroformate. Vortex the vial for 10 s and sonicate it for 1 min. (2) Add 300 μl of n-hexane into the injection vial and vortex it in 2,000 r.p.m. for 60 s. (3) After centrifugation at 12,000 r.p.m. for 5 min at 4 °C, transfer 250 μl of the obtained n-hexane layer into a new injection vial. (4) Add another 200 μl of n-hexane into the original injection vial and vortex it in 2,000 r.p.m. for 60 s. (5) After centrifugation at 12,000 rpm for 5 min at 4 °C, transfer 200 μl of the n-hexane layer into the new injection vial. (6) Add 10 mg of anhydrous sodium sulfate into the obtained 450 μl of n-hexane layer and vortex it for 10 s. Then analyze it using a GC-MS system.

Later, standards derivation was conducted as follows: (1) Into an injection vial, add 300 μl of mixed standards solution and 500 μl of NaOH solution (0.005 mol/L). (2) Add 500 μl of propanol/pyridine (3:2, v/v) and 100 μl of propyl chloroformate into the injection vial. Vortex the vial for 10 s and sonicate it for 1 min. (3) Add 300 μl of n-hexane into the injection vial and vortex it at 2,000 rpm for 60 s. (4) After centrifugation at 12,000 rpm for 5 min at 4 °C, transfer 250 μl of the obtained n-hexane layer into a new injection vial. (5) Add an additional 200 μl of n-hexane into the original injection vial and vortex it at 2,000 rpm for 60 s. (6) After centrifugation at 12,000 rpm for 5 min at 4 °C, transfer 200 μl of the n-hexane layer into the new injection vial. (7) Add 10 mg of anhydrous sodium sulfate into the obtained 450 μl of n-hexane layer and vortex it for 10 s. Then analyze the mixture using a GC-MS system. The detailed information of GC-MS procedure was displayed in Supplementary Material.

### Statistical analysis

Statistical analysis was performed using GraphPad Prism version 10 (GraphPad Software Inc., United States). All the data are presented as the means ± standard errors of the mean (SEMs). Student's *t*-test or Mann–Whitney *U* test was used to measure the difference in normally or non-normally distributed data, respectively. The Shapiro–Wilk test was performed to examine the normality. Pearson correlation analysis or Spearman correlation analysis was conducted as appropriate. Levene's test was used to assess the variances between the two groups, and if the variances were not similar, an adjusted *p*-value was used. The taxonomic distributions of OTUs were summarized to calculate the relative abundances of gut microbiota at different levels. Four parameters (Shannon, Simpson, phylogenetic diversity, and Chao) were used to assess the alpha diversity. Beta diversity, which measures the dissimilarity between samples, was assessed using principal coordinate analysis (PCoA). The Random Forest algorithm was applied to identify key discriminatory OTUs. Furthermore, the linear discriminant-analysis effect size (LEfSe) was used to identify the dominant bacterial taxa in both the control and aged rats. A *p*-value less than 0.05 was considered significant, and the Benjamini and Hochberg False Discovery method was used for multiple testing corrections.

## Results

### The induction rate of atrial fibrillation (AF) significantly increased, while atrial conduction velocity decreased, conduction dispersion increased, and heart rate variability (HRV) decreased in aged rats

**Figure 1:**
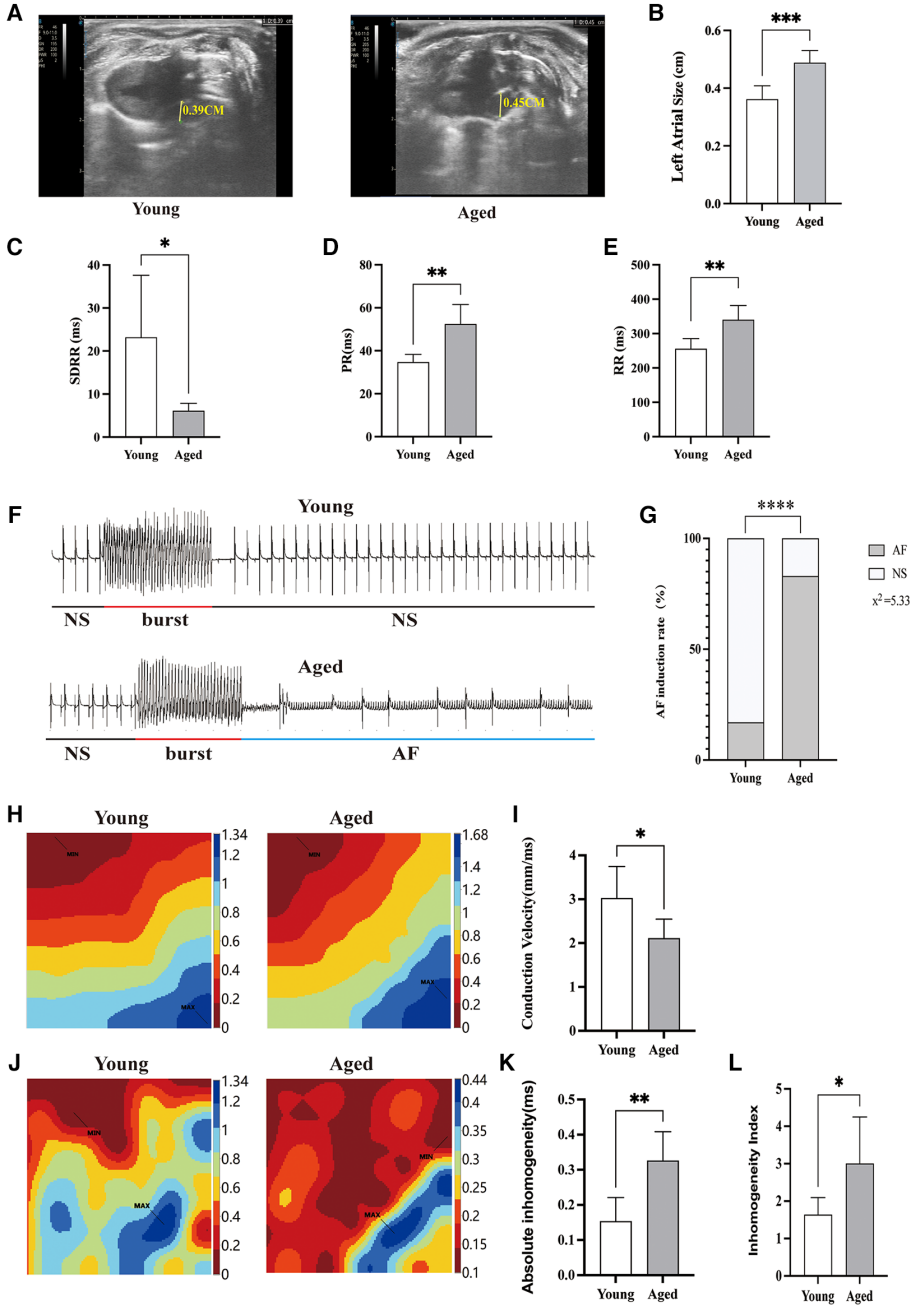
AF inducibility rate and atrial electrophysiology in aged rats. (**A**) Representative echocardiographic parasternal long axis views of rats. Left atrial diameter in rats (yellow line segment). (**B**) The left atrial diameter size was significantly increased in the aged rats group compared to the young rats group. (**C**) Decreased heart rate variability in aged rats; (**D**,**E**) the PR interval and RR interval of aged rats are prolonged. (**F**) Typical ECG example illustrating the induction of AF; (**G**) increased incidence of AF in aged rats; (**H**) representative activation map; (**I**) reduced atrial conduction velocity in aged rats; (**J**) representative inhomogeneity maps; (**K**) Increased absolute inhomogeneity (P95-5); (**L**) increased inhomogeneity index (P95-5/P50). MAX and MIN represent the maximum and minimum values of inhomogeneity, respectively; SDRR, standard deviation of RR intervals; NS, normal sinus rhythm; AF, atrial fibrillation; Data obtained from *n* = 6 hearts. *P < 0.05, **P < 0.01.

Echocardiographic assessment indicated that the left atrial diameter size was significantly increased in the aged rat group compared to the young rat group (Figures 1A, B**)**. Time-domain analysis of HRV was conducted to assess its overall regulatory function on cardiac autonomic nerves. The study results revealed that, in comparison with young rats, the SDRR value of aged rats exhibited a significant reduction (*P* < 0.01), indicating decreased heart rate variability and disrupted autonomic nervous system (ANS) function (Figure 1C). The study found a significant prolongation of PR and RR intervals in aged rats compared to young rats (*P* < 0.01) (Figures 1D, E**)**. These findings indicate decreased heart rate variability and disrupt autonomic nervous system (ANS) function in aged rats. Burst stimulation was conducted on the hearts to induce AF (Figure 1F). The findings demonstrated that among the young rats' hearts, 1 out of 6 developed AF. In contrast, AF was observed in 5 out of 6 aged rats' hearts. These results indicated a significant increase in the induction rate of AF in aged rats (Figure 1G).

Slowing of atrial conduction velocity, increased conduction dispersion, and the formation of reentry all play crucial roles in triggering and sustaining AF (16). Representative activation transmission maps of the left atrial surface for each group are depicted in Figure 1H. In young rats, propagation occurs rapidly from the first excited electrode to the last excited electrode. In contrast, signal progression in aged rats was slower compared to young rats, and this alteration was accompanied by an elevation in total activation time and a reduction in conduction velocity (Figure 1I). Representative inhomogeneity maps were obtained in both groups as illustrated in Figure 1J. In the aged rat group, P95-P5 and P95-P5/P50 values were significantly increased, indicating an increase in the absolute inhomogeneity and inhomogeneity index among aged rats (Figures 1K, L).

These observations suggest that aging may lead to dysfunction of the ANS, which could potentially contribute to the development of AF in the elderly. This highlights the importance and potential applicability of our animal model in studying the underlying mechanisms of AF in older individuals.

### Dysbiosis of gut microbiota in aged rats

**Figure 2:**
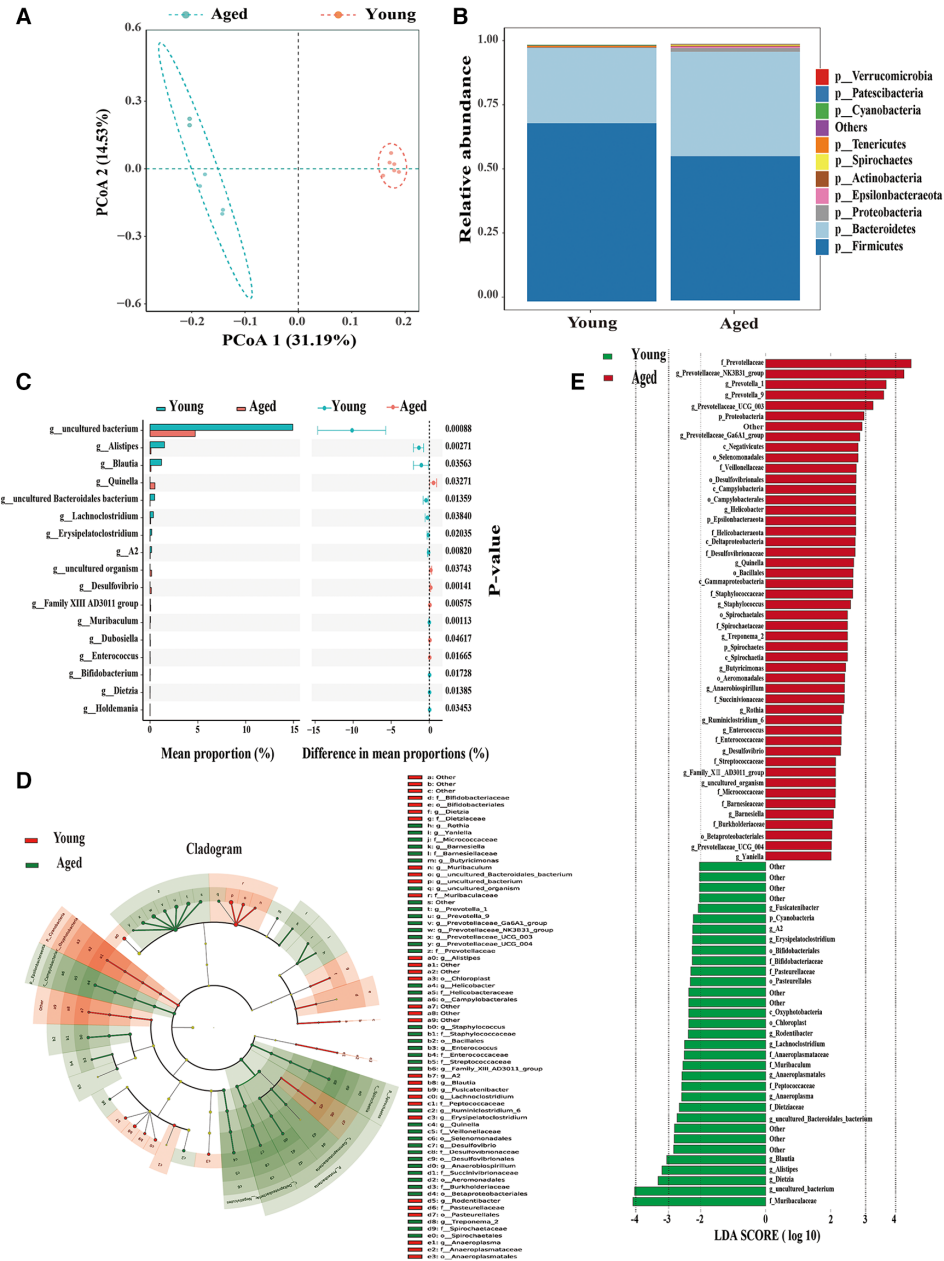
Significant alterations in gut microbiota in aged rats compared to young rats. (**A**) PCoA demonstrates a noticeable difference in the composition of gut microbiota between the two groups; (**B**) phylum-level changes in the young and aged groups; (**C**) identification of 17 differential bacterial taxa on the genus level; (**D**,**E**) identification of dominant bacterial taxa in different groups using LEfSe. Six rats were included in each group.

We profiled the gut microbiota from young and aged rats by analyzing the DNA sequences encoding the 16S rRNA gene. The principal coordinates analysis (PCoA) of Unweighted UniFrac distances (β-diversity) among aged and young rats showed significant separation in microbiota community structures due to aging along both the first principal coordinate (PC1 axis) and the second principal coordinate (PC2 axis) (Figure 2A).

At the phylum level, Bacteroidetes increased while Firmicutes decreased with aging (Figure 2B). Previous research has indicated a significantly decreased ratio of Firmicutes to Bacteroidetes (F/B ratio), which serves as an indicator of gut microbial dysbiosis in aging vs. young rats (12). Consistent with previous studies, we also observed a decrease in Firmicutes and an increase in Bacteroidetes in aged rats, resulting in a decreased F/B ratio. These findings indicate a significant difference in the composition of the gut microbiome community between young and aged rats. Further analysis revealed that 17 differential bacterial taxa at the genus level were significantly altered in aged rats (Figure 2C). Therefore, aging is closely associated with the disorder of intestinal microbiota.

To identify dominant bacterial taxa in different groups, we used LEfSe, a new method for metagenomic biomarker identification through class comparison. In total, we found 81 bacterial taxa with statistically significant and biologically consistent differences (LEfSe LDA>2, *P* < 0.05) (Figures 2D, E). These bacterial taxa represent key phylotypes that contribute to the differences in gut microbiota between young and aged rats. Among these, 48 bacterial taxa were most abundant in young rats, while 33 bacterial taxa were most abundant in aged rats. The most differentially abundant bacterial taxa (LDA>3) in young rats belonged to the genera Blautia, Alistipes, and Dietzia. The most differentially abundant bacterial taxa (LDA>3) in aged rats belonged to the genera Prevotellaceae-NK3B31-group, Prevotella-1, and Prevotella-9.

Our findings support the suggestion that the increased susceptibility to AF is closely associated with the development of gut dysbiosis during the aging process.

### Differentially expressed SCFAs levels in aged rats

**Figure 3:**
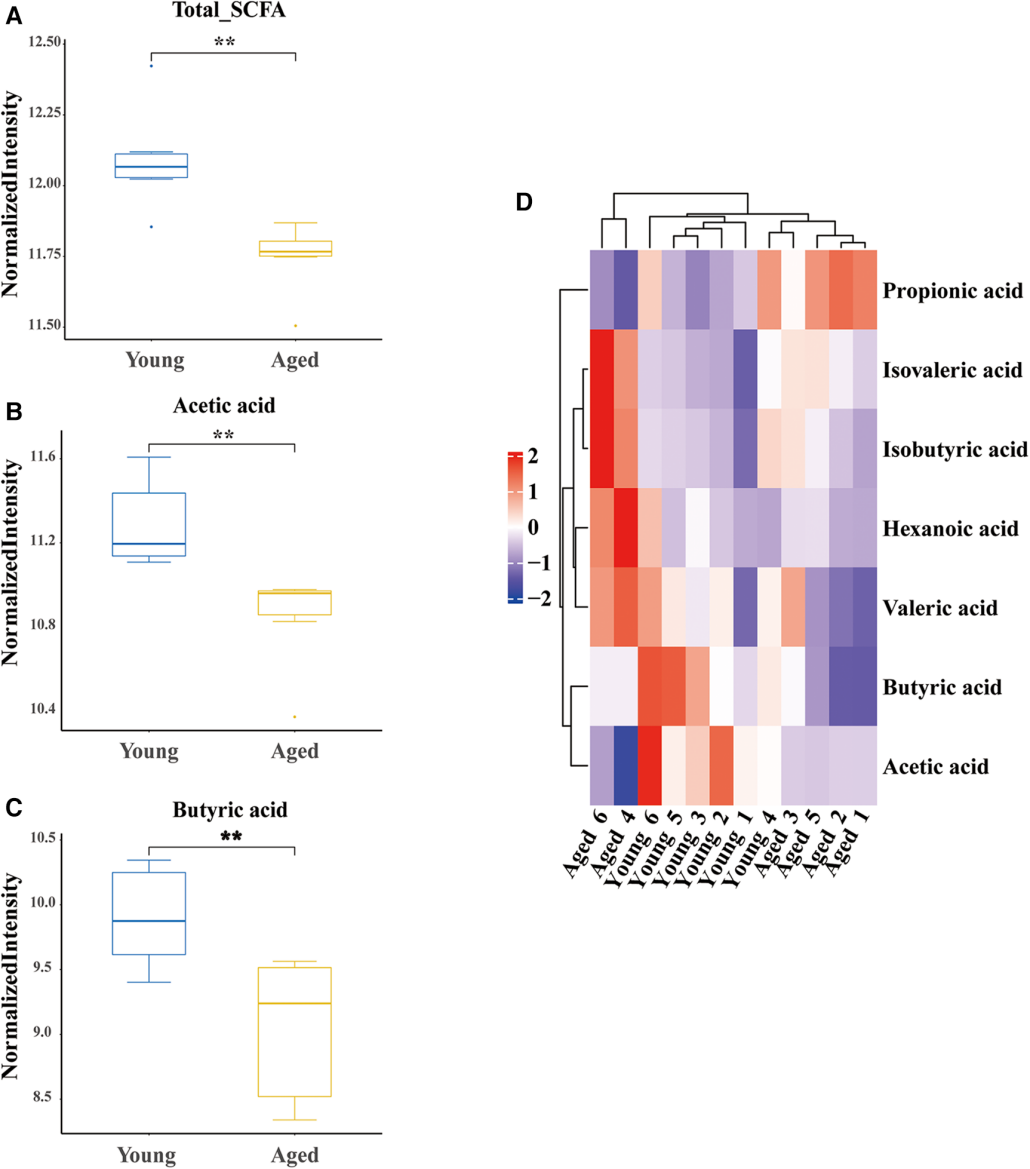
Differential identification of SCFAs. (**A**) Decreased total SCFAs in aged rats; (**B**) decreased acetic acid in aged rats; (**C**) decreased butyric acid in aged rats; (**D**) hot map. SCFAs short chain fatty acids; six rats were included in each group. *P < 0.05, **P < 0.01.

SCFAs are a major class of key bacterial metabolites that are important for human health. In this study, we measured the levels of seven SCFAs (acetic acid, propionic acid, butyric acid, pentatonic acid, hexanoic acid, isobutyric acid, and isovaleric acid) in fecal samples of young and aged rats. We found that three major SCFAs (acetic acid, butyric acid, and isovaleric acid) were successfully identified. The total levels of SCFAs were significantly decreased in aged rats (*P* = 0.005, Figure 3A). Specifically, the levels of acetic acid (*P* = 0.006, Figure 3B) and butyric acid (*P* = 0.001, Figure 3C) were significantly decreased in the aged group, while the level of isovaleric acid was significantly increased (*P* = 0.028, Figure 3D).

### Significant correlation between gut microbiota dysbiosis, SCFAs, and heart rate variability in aged rats

**Figure 4:**
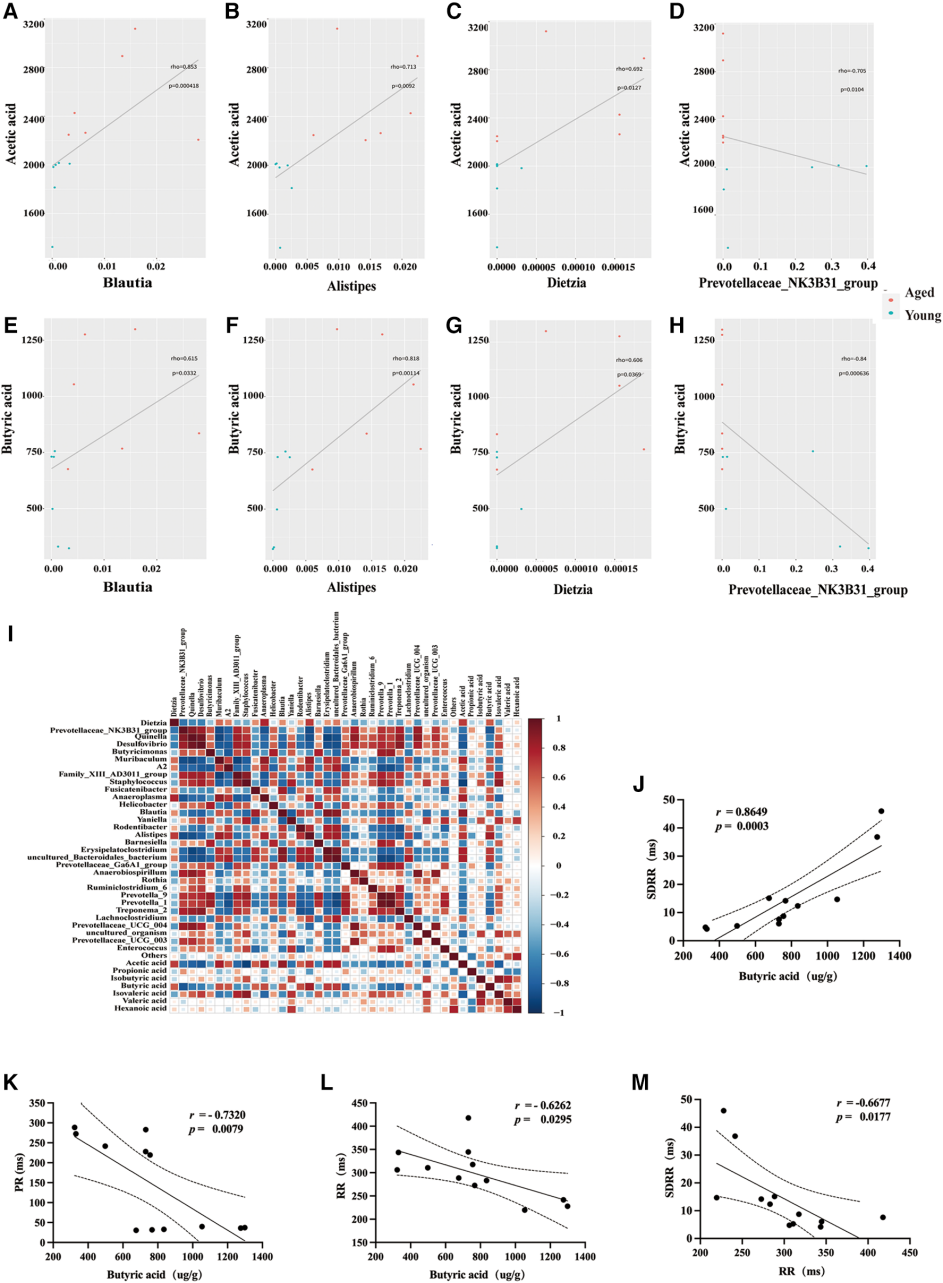
Correlations between differential SCFAs, HRV, and bacterial taxa. (**A**–**H**) Scatter plots were used to visually represent the relationships between differential SCFAs and bacterial taxa. (**I**) Heatmap showing the correlations between differential SCFAs and bacterial taxa. (**J**–**L**) Correlations between butyric acid and HRV. (**M**) Correlations between RR and SDRR. SCFAs, short chain fatty acids; HRV, heart rate variability; SDRR, standard deviation of RR intervals.

In this study, we employed the Spearman statistical method to conduct a correlation analysis on changes in the time-threshold indicator SDRR of intestinal flora, SCFAs, and HRV. The results demonstrated a significant positive correlation between acetic acid and three bacterial genera (Blautia, Alistipes, and Dietzia) in young rats: Blautia genus (*r *= 0.853, *P* = 0.000418) (Figure 4A), Alistipes genus (*r *= 0.713, *P* = 0.0092) (Figure 4B), and Dietzia genus (*r *= 0.692, *P* = 0.0127) (Figure 4C). Notably, there was a significant negative correlation between acetic acid and Prevotellaceae in aged rats (*r *= −0.705, *P* = 0.0104) (Figure 4D). Furthermore, our study revealed a significant positive correlation between Butyric acid and three distinct probiotics in young rats: Blautia genus (*r *= 0.853, *P* = 0.000418) (Figure 4E), Alistipes genus (*r *= 0.818, *P* = 0.00114) (Figure 4F), and Dietzia genus (*r *= 0.606, *P* = 0.0369) (Figure 4G). Conversely, a significant negative correlation was observed between Butyric acid and Prevotellaceae bacteria in aged rats (*r *= −0.84, *p* = 0.000636) (Figure 4H). The heatmap presented in the lower left corner illustrates the correlation between differential SCFAs and bacterial taxa (Figure 4I). Additionally, we performed a correlation analysis between the measured SCFAs (Butyric acid) and HRV (SDRR,PR,RR). The study revealed a significant positive correlation between Butyric acid levels and SDRR (*r* = 0.8649, *P* = 0.0003), and a significant negative correlation between Butyric acid levels and PR interval (*r* = −0.7320, *P* = 0.0079), as well as RR interval (*r* = −0.6262, *P* = 0.0295). Furthermore, a significant negative correlation was observed between RR interval and SDRR (*r* = −0.6677, *P* = 0.0177) (Figures 4J–M).

These results further support the potential involvement of SCFAs (metabolites of gut microbiota) in the development of AF in the elderly, potentially mediated through the modulation of ANS function.

## Discussion

### Major findings

**Figure 5:**
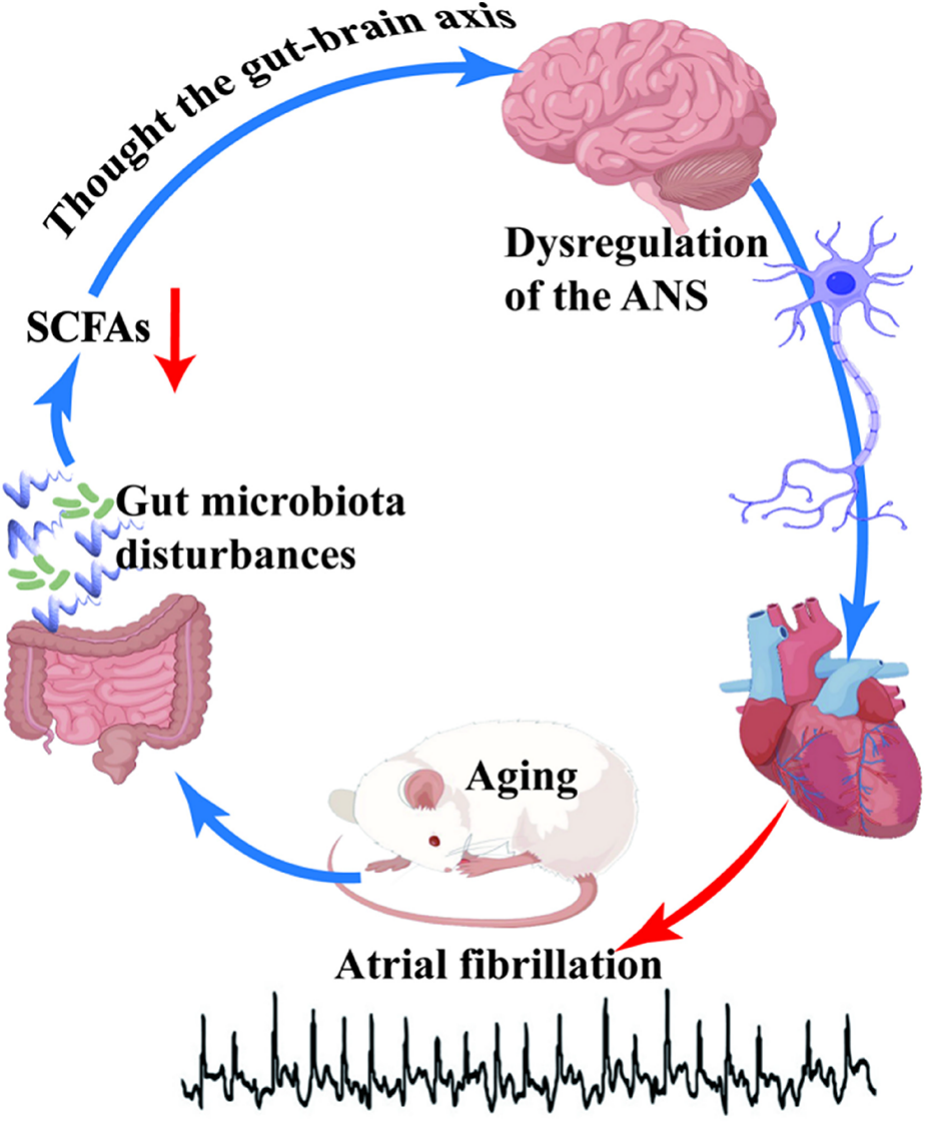
The schematic representation in this study highlights the correlation between disordered gut microbiota and age-related atrial fibrillation. Aging induces dysbiosis of the gut microbiota, subsequently leading to a reduction in the levels of short-chain fatty acids (SCFAs), which are vital metabolic byproducts of the gut microbiota. SCFAs exert their regulatory effects on the autonomic nervous system (ANS) via the gut-brain axis. A decline in SCFAs levels disrupts ANS homeostasis, thereby enhancing susceptibility to atrial fibrillation in aged rats.

In our study, we investigated the potential relationship between age-related gut microbiota disturbances, reduction of short-chain fatty acids (SCFAs), and autonomic nervous system (ANS) dysfunction (Figure 5), and their contribution to the occurrence and progression of atrial fibrillation (AF) in the elderly. Our findings revealed a significant increase in the induction rate of AF in aged rats, accompanied by slowed atrial conduction velocity, increased conduction dispersion, and reduced heart rate variability (HRV). Moreover, we observed disordered gut microbiota and decreased SCFAs levels in aged rats, which were significantly correlated with the reduced HRV. These results strongly support our research hypothesis that the disruption of age-related gut microbiota and decrease in SCFAs may contribute to the dysfunction of the ANS, thereby promoting the development and progression of AF.

### Increased incidence of AF in aged rats, slowed atrial conduction velocity, increased conduction dispersion, and decreased HRV

The occurrence of AF is positively correlated with increasing age (3). The mechanism by which aging causes AF involves changes in cardiac electrophysiology, cardiac structure, inflammation, and oxidative stress, and the ANS (17). As individuals age, the function of ion channels in atrial myocytes changes, leading to prolonged action potential duration and increased electrical activity instability. These changes may contribute to the slowing of atrial conduction velocity and increased susceptibility to AF in older adults (18). Additionally, aging can lead to structural changes in cardiac tissue, such as cardiomyocyte hypertrophy, interstitial fibrosis, and atrial dilation, which further affect atrial conduction velocity and conduction dispersion, creating favorable conditions for the occurrence of AF (19). Gan et al. (18) discovered that as individuals age, the conduction velocity of atrial electrical impulses decreases, along with a decrease in the expression of intrinsic L-type calcium current (I_ca−L_), Ca^2+^ exchange proteins, and other substances in atrial myocytes. These changes result in action potential firing, conduction, and morphological alterations based on the formation of I_ca−L_, thereby increasing the likelihood of AF. Our study's results demonstrate that aged rats have a significantly higher induction rate of AF, slower atrial conduction velocity, increased conduction dispersion, and decreased HRV. These findings align with previous studies and further support the impact of aging on cardiac electrophysiology.

The alterations in both size and function of the left atrium are closely associated with the development and progression of atrial fibrillation (AF). Multiple studies have indicated a correlation between increased left atrial size and the risk of AF onset and maintenance (20, 21). Our findings demonstrate a significant enlargement of the left atrium in rats with atrial fibrillation compared to those with normal sinus rhythm. These results are consistent with previous research, emphasizing the prognostic value of left atrial size in AF. Identifying left atrial size as a potential biomarker for AF holds significant clinical implications.

HRV is a widely used physiological variable for non-invasively assessing cardiac autonomic activity by measuring changes in ANS (22). In patients with AF, HRV tends to decrease to varying degrees due to the degradation of ANS function. The emergence of non-invasive techniques, such as HRV, allows for a more detailed assessment of the role of the ANS in AF (23). In HRV time domain analysis, Standard deviation of RR intervals (SDRR) encompasses various frequency components of HRV, providing an overall assessment of HRV and the regulation of the ANS (24). Esler et al. (6) discovered that as age increases, there is an increase in the conversion of central sympathetic norepinephrine, which can activate the sympathetic nerve. This leads to changes in plasma norepinephrine levels, β-adrenoceptor sensitivity, and receptor signaling pathways in patients with AF, ultimately resulting in reduced HRV. Other related studies have also highlighted the crucial role of the ANS in the development, spread, and complexity of AF (23), which aligns with our findings. In our study, we observed changes in cardiac electrophysiology and dysfunction of ANS in aged rats. Aging causes a slowdown in atrial conduction velocity, an increase in conduction dispersion, and a decrease in HRV in aged rats, thereby increasing the incidence of AF. These results provide new insights and evidence for studying the mechanism of AF in the elderly.

### Gut microbiota disturbances and reduction of SCFAs in aged rats

The gut microbiota, being the most important and largest microecological system in the human body, experiences changes and imbalances in type and quantity that can affect the body's physiological functions (25). Previous studies have indicated that although there are similarities in the composition of gut microbiota between rats and humans, there are still some differences in terms of quantity and species (26). The human gut microbiota exhibits significant variations among individuals, which may be influenced by factors such as diet, lifestyle, and genetics (27). In contrast, the gut microbiota of rats is relatively more stable and heavily influenced by host genetic factors (26). Firmicutes and Bacteroidetes are the dominant phyla in the human gut microbiota (27). The major bacterial groups in rat gut microbiota include Firmicutes, Bacteroidetes, Actinobacteria, Proteobacteria, and Verrucomicrobia. Among these, Firmicutes and Bacteroidetes are the two most abundant phyla, comprising most of the gut microbiota (28). Gut microbiota dysbiosis plays a significant role in the development of age-related diseases (29). As age and the occurrence of aging-related diseases, the gut microbiota undergoes significant changes. For instance, Zhang et al. (12) used 16sRNA gene detection technology and metabolomics to analyze the gut microbiota of old AF rats, revealing significant changes in its structural composition. In our study, we discovered that the gut microbiota of aged rats was disturbance, potentially due to aging. We conducted 16S sequencing on the gut microbiota of aged rats, and the PCoA results demonstrated a significant separation in the bacterial community structure as rats aged.

The ratio between Firmicutes and Bacteroidetes can serve as an indicator of gut microbiota disturbance, which decreases with age (12, 30). Our analysis at the bacterial phylum level revealed an increase in Bacteroidetes and a decrease in Firmicutes with age, indicating disruption in the gut microbiota of aged rats. These findings are consistent with previous studies. A study utilizing high-throughput sequencing technology examined the characteristics of gut microbiota in the elderly population. The results revealed that the elderly had a higher proportion of Bacteroidetes, whereas the young had a higher proportion of Firmicutes (31). Zhang et al. (12) conducted animal experiments and discovered that aging rats exhibited an imbalanced gut microbiota, characterized by a decrease in Bifidobacterial and Firmicutes, and an increase in Bacteroidetes and Enterobacteriaceae. Our study further demonstrated that the gut microbiota of aged rats was disrupted, specifically showing a decrease in Firmicutes. Subsequent analysis at the genus level revealed significant changes in 17 differential bacterial groups in aged rats. Utilizing the LEfSe analysis method, we identified 81 bacterial taxa with significant differences. These findings support the alterations in bacterial community structure, both at the phylum and genus levels, and align with existing research on the gut microbiota in relation to aging. Therefore, our results suggest that changes in gut microbiota composition may be associated with the aging process.

SCFAs, including acetate, propionate, and butyrate, are crucial metabolites of gut microbiota (32). SCFAs are closely associated with age-related diseases (33). As individuals age, there is a general disruption in the diversity of gut microbiota resulting in a relative decrease in the bacterial flora responsible for SCFA production, consequently reducing SCFAs levels (34). The findings of this study indicate a significant reduction in the total level of SCFAs, particularly in acetic acid and butyric acid levels. These results align with previous studies. Claesson et al. (33) analyzed fecal samples from 161 individuals aged over 65 years and 9 adults aged 28–46 years, observing a higher proportion of Firmicutes (the main phylum responsible for SCFAs) in the older group compared to the younger group. More than 65% of subjects in the older group exhibited an imbalance in the Firmicutes/Bacteroidetes (F/B) ratio in their microbial communities. Kim et al. (34) demonstrated that SCFAs-producing Clostridium bacteria in the intestines of the elderly are often reduced, while aerobic and pathogenic bacteria increase. These findings emphasize the role of SCFAs in age-related diseases and suggest the potential for clinical interventions targeting the gut microbiota to modulate SCFAs levels.

Probiotics, live microorganisms that confer health benefits when administered in adequate amounts, have been shown to modulate the gut microbiota by promoting the growth of beneficial bacteria and suppressing the growth of potentially harmful ones (35, 36). Understanding the theoretical basis for considering probiotics and SCFAs interventions in the context of AF is crucial. The gut microbiota has emerged as a key player in cardiovascular health, with growing evidence suggesting its involvement in AF development (12). Butyric acid is another extensively studied intestinal microbiota metabolite. Jiang et al. (37) utilized sodium butyrate, a SCAFs, as a potential therapeutic intervention in myocardial infarction in rats and found that it could improve cardiac function and electrical stability, reduce post-myocardial infarction cardiac dysfunction and arrhythmias.

### Correlation between gut microbiota/SCFAs and HRV

Aging has been found to be associated with a decline in the levels of SCFAs produced by intestinal microorganisms, which in turn affects the functioning of the central nervous system and interacts with the regulation of ANS activity (38) The results of our study demonstrate a significant correlation between gut microbiota disorder, reduction in SCFAs, and a decrease in HRV in aged rats. Our experimental findings indicate a positive relationship between SCFAs levels and the presence of probiotic bacteria, specifically Alistipes and Blautia, in young rats. These results align with previous research. Lee et al. (38) investigated the SCFAs content in the intestines of aged mice and observed that an imbalance between probiotic and pathogenic bacteria resulted in reduced SCFAs levels. These findings strongly support the notion that there is a close connection between aging-related gut microbiota and SCFAs, suggesting that clinical interventions targeting the gut microbiota and influencing SCFAs levels may hold promise for the treatment of age-related diseases.

We observed a decrease in SCFAs levels in aged rats, which was found to be closely associated with ANS dysfunction. Previous studies have demonstrated that SCFAs can impact various bodily functions through multiple potential pathways, including endocrine, immune regulatory, vagal, and humoral pathways (30) SCFAs activate receptors such as GPR41 and GPR43 in intestinal vagal afferent nerves, thereby regulating the activity of the host's sympathetic and parasympathetic nervous systems through the gut-brain axis. This indirect regulation of cardiac autonomic nerves by SCFAs contributes to cardiovascular protection (39–41). Disruption of intestinal microbial homeostasis and compromised intestinal barrier integrity can result in elevated blood SCFAs concentrations, thereby intensifying the direct effects of SCFAs on cardiac autonomic nerves (42, 43). While an increase in central sympathetic outflow associated with SCFA has been described, the effects of SCFA on the intrinsic cardiac nervous system, which plays a pivotal role in cardiac homeostasis as well as in the onset and maintenance of AF (44), have never been specifically assessed but might play a significant role. Kimura et al. (14) discovered that dietary fiber consumption leads to the production of SCFAs through microbial metabolism in the colon, which in turn regulate sympathetic nerves. The authors specifically found that propionate acts on the GPR41 receptor in autonomic ganglia, directly influencing autonomic nervous function. In a study by LAL et al. (45), sodium butyrate was perfused into the intestines of anesthetized male rats, resulting in a distinctive response in mesenteric afferent nerves and the activation of vagal afferent nerves. Importantly, this response occurred independently of vagal afferent nerve activation, as demonstrated by the disappearance of the phenomenon following vagotomy. The direct action of the cholecystokinin receptor (CCK-A) on vagal afferent nerves was also observed, which further supports the regulatory role of SCFAs, the primary metabolites of gut microbiota, on the ANS.

HRV is an important indicator that reflects the functional status of the ANS. In our study, we found a negative correlation between resting heart rate (RR interval) and SDRR, consistent with prior research (46). This further emphasizes our findings. As age increases, both RR interval and SDRR are expected to increase, but we observed an unexpected decrease in SDRR. This phenomenon can be partly attributed to the non-linearity of neural regulation cycle length, resulting in inherent rate dependency of autonomic indices including SDRR, a notion supported by Zaza et al. (46). There are some limitations in assessing HRV indices using time domain measures, and it is necessary to evaluate normalized frequency domain indices, which are less affected by inherent rate dependency. Given that our study is correlational and lacks intervention confirmation, it only provides a direction for further research.

SCFAs play a crucial role in human health as they regulate the immune system, energy metabolism, and intestinal mucosal barrier. In our study, we conducted a correlation analysis between all detected SCFAs and HRV in aged rats. The results showed a significant correlation between SCFAs levels and HRV, suggesting that SCFAs may mediate the occurrence and development of AF in the elderly by regulating autonomic nervous function. These findings are consistent with previous studies. For instance, Yu et al. (10) analyzed HRV and norepinephrine (NE) detection and found that butyrate significantly inhibited autonomic imbalance induced by ischemia/reperfusion (I/R), but this effect was not observed in the vagotomy group. Furthermore, butyrate treatment was found to inhibit neural activity in the paraventricular nucleus (PVN) and superior cervical ganglion (SCG), and these effects disappeared after vagotomy. Additionally, we observed a negative correlation between resting heart rate and SDRR, indicating reduced heart rate variability with higher heart rates. Our analysis also revealed a negative correlation between butyric acid and PR/RR intervals, further supporting our hypothesis that reduced SCFAs with aging may disrupt cardiac pacing and conduction via the autonomic nervous system, increasing susceptibility to AF. Therefore, our findings are in line with previous research, highlighting the important role of gut microbiota and SCFAs in the pathogenesis of AF in the elderly. The disturbance of age-related gut microbiota and reduction of SCFAs may contribute to the dysfunction of the ANS, thus promoting the occurrence and progression of AF.

## Limitation

This study acknowledges limitations, including a small sample size affecting statistical power and limited exploration of intervention strategies involving probiotics or short-chain fatty acids. Future research should prioritize validating these findings through human studies.

## Conclusion

In this study, we demonstrated that the increased susceptibility to AF in aged rats is closely related to reduced SCFA levels caused by age-induced gut microbiota disturbances. In addition, we found a relationship between reduced SCFA levels and autonomic nervous system dysfunction. Based on our findings, we can preliminarily hypothesize that SCFAs, which are metabolites of gut microbiota, may play a role in the occurrence and progression of AF in the elderly by regulating the ANS. Additionally, our study suggests that supplementation of SCFAs could be a potential approach to modulating neurotransmitter networks. This is an innovative finding in the understanding of the function of SCFAs. Our research provides compelling evidence that gut microbiota and SCFAs dysregulation are associated with the pathophysiology of age-related AF. This highlights the significance of studying the etiology of AF and its potential implications for prevention and treatment.

## Data Availability

The raw data supporting the conclusions of this article will be made available by the authors, without undue reservation.
