# Supplementary material for: Impact of age-related gut microbiota dysbiosis and reduced short-chain fatty acids on the autonomic nervous system and atrial fibrillation in rats
Source: Front Cardiovasc Med. 2024 Jun 12;11:1394929. doi: 10.3389/fcvm.2024.1394929 (PMC11199889; doi:10.3389/fcvm.2024.1394929)
Supplement: Supplementary file 1 [file Datasheet1.pdf]

Supplementary Material

Journal: Frontiers in Cardiovascular Medicine, section Cardiac Rhythmology

Article type: Original Research

Manuscript title: Impact of Age-related Gut Microbiota Dysbiosis and Reduced Short-chain Fatty Acids on the Autonomic Nervous System and Atrial Fibrillation in Rats Authors

Manuscript ID: 1394929

Authors: Li Liu, Ying qi Yi, Rong Yan, Rong Hu, Wei hong Sun, Wei Zhou, Hai yan Zhou, Xiao yun Si, Yun Ye, Wei Li and Jingjing Chen

Submitted on: 02 Mar 2024

Interactive review started on: 06 Apr 2024

Supplementary materials for the Methods section.

1.16S rRNA Gene Sequencing

1.1 Stool Samples Collection

Fecal samples were collected from rats and immediately frozen with liquid nitrogen for 15 min, then stored at −80 ˚C until analysis.

1.2 DNA extraction and PCR amplification

DNA was extracted from the stool samples and 16S rRNA amplicon sequencing were performed using the NovaSeq PE250 sequencing platform, according to the manufacturer's instructions. Data were collected as previously described in Chen et al. (2020). Specifically, Genomic DNA of the samples were extracted by SDS-CTAB method, and the purity and concentration of DNA were detected by agarose gel electrophoresis. Appropriate amount of DNA was taken and diluted to 1ng/µl in sterile water. Using diluted genomic DNA as a template, the V4 region of the bacterial 16S rRNA was amplified by PCR with the specific primers 515F(5′-GTGCCAGCMGCCGCGGTAA-3′) and 806R(5′-GGACTACHVGGGTWTCTAAT-3′) labeled in a 12 bp barcode. Using high-fidelity PCR Master Mix with GC Buffer (Phusion®; New England Biolabs, Ipswich, MA, USA) and high-fidelity enzyme to ensure amplification efficiency and accuracy.

2. Sequencing and data processing

Reads for each sample were demultiplex from the raw data according to Barcode sequence and PCR primer sequence. Original tags data (raw tags) were formed by splicing the reads from each sample using FLASH (V1.2.7, http://ccb.jhu.edu/software/FLASH/). The spliced raw tags were strict filtered to get high-quality clean tags according to published protocols (Qiime V1.9.1, http://qiime.org/scripts/split_libraries_fastq.html). Clean tags detected chimeric sequences by comparing with the species annotation database (https://github.com/torognes/vsearch/) and finally removed the chimeric sequences to obtain the final effective tags. Operational Taxonomic Units (OTUs) were clustered against the Uparse software (Uparse V7.0.1001, http://www.drive5.com/uparse/) at 97% identity for all effective tags. The representative sequence of the OTUs was analyzed by mothur method and SILVA138 (http://www.arb-silva.de/) SSU rRNA database. MUSCLE program (Version 3.8.31, http://www.drive5.com/muscle/) was used for fast multi-sequence alignment to obtain the phylogenetic relationships of all OTUs. Finally, the data of all samples were homogenized, and the sample with the least amount of data was taken as the standard for homogenization. The subsequent Alpha and Beta diversity analysis were based on the homogenized data.

3. Alpha diversity analysis

We analyzed within-community microbial diversity by using alpha diversity analysis. Qiime v1.9.1 was used to calculate Observed species, Chao1, ACE, Shannon, Simpson, PD_whole_tree indexes. R version was used to plot the rarefaction curve, rank abundance curve and species accumulation boxplot. The difference analysis between AF group and SR group of alpha diversity indexes were conducted by t -test and Wilcox test.

4. Beta diversity analysis

We analyzed the dissimilarities in the microbiomes by using beta diversity analysis. Qiime v1.9.1 was used to calculate unweighted unique fraction metric (Unifrac) between AF group and SR group. Principal co-ordinates analysis (PCoA) and non-metric multidimensional Scaling (NMDS) diagrams were drawn using Rv2.15.3. We performed PCoA by using WGCNA, STATS and GGplot2 packages of R and performed NMDS analysis by using vegan package of R. Analysis of similarities (Anosim),

multi-response permutation procedures (MRPP) and Adonis (permutational multivariate analysis of variance) were analyzed by using the Anosim function, MRPP function and Adonis function of R vegan package respectively. The difference analysis in the unweighted Unifrac between the AF and SR groups were conducted by t -test and Wilcox test in R software.

5. **LEfSe analysis**

LEfSe software was used for the linear discriminant analysis (LDA) effect size (LEfSe) analysis and the threshold of LDA score (log10) was set to 4. Permutation tests were performed between groups at various classification levels (Phylum, Class, Order, Family, Genus and Species) by using the metastats analysis of R software, and P-values were obtained. Then, the Benjamini and Hochberg false discovery rate method was used to correct P-values and obtained q-values (Edgar, 2004). The difference analysis between the AF and SR groups were also using the t -test and Wilcox test in R software.Additionally, Spearman correlation was used to associate abundant differential taxa with SCFA.

Short-Chain Fatty Acids (SCFAs) Identification

1. **Instruments**

The GC-MS system mainly consisted of gas mass spectrometer (Agilent Model 7890-5977， USA), vortex mixer (QL-866, China) and refrigerated centrifuge (Xiangyi, China). Agilent FFAP capillary GC column (30 m × 0.25

mm ID × 0.25 µm).

2. **Preparation of Standard Solutions**

Weigh the standard substances, including acetic acid, propionic acid, butyric acid, isobutyric acid, valeric acid, isovaleric acid, and hexanoic acid. Prepare a stock solution with a concentration of 50mg/mL by dissolving these substances in ethyl acetate, and store it at -20°C. Further dilute the stock solution to obtain standard solutions with different concentrations.

3. Metabolite Extraction

Take approximately 100mg of the sample and transfer it to a 2 mL centrifuge tube. Add 100 μL of 20% phosphoric acid for reconstitution and shake vigorously for 2 minutes. Then, add 500 μL of ethyl acetate solution containing an internal standard (4-methylvaleric acid) at a concentration of 50 μg/mL and mix well for 1 minute. Centrifuge the mixture at 14000 g for 20 minutes at 4°C. After allowing it to settle for 30 minutes, carefully collect the supernatant, remove any remaining water using anhydrous sodium sulfate, and proceed with GC-MS analysis. Inject 1 μL of the extracted solution into the system using a split ratio of 10:1.

4. Gas Chromatography-Mass Spectrometry Analysis

Column: FFAP (30m × 250μm × 0.25μm); Injection volume: 1μL; Injection temperature: 250°C; Split ratio: 10:1; Carrier gas: Helium (99.999% purity); Flow rate: 1mL/min; Oven temperature: 100°C held for 1 min, ramped at 5°C/min to 160°C, not held, ramped at 80°C/min to 250°C, held for 6 min; Interface temperature: 260°C; Ion source temperature: 230°C; Quadrupole temperature: 150°C; Ionization mode: EI, 70eV; Detector voltage: 1000V; Scan mode: Selected Ion Monitoring (SIM); Mass range: 20~350; SIM parameters: acetic acid at 4.36 min, m/z 60; propionic acid and isobutyric acid at 5.64 min and 6.06 min, m/z 73.00; butyric acid, isovaleric acid, and valeric acid at 7.05 min, 7.48 min, and 8.98 min, m/z 60; hexanoic acid at 10.98 min, m/z 60.00; internal standard 4-methylvaleric acid at 10.19 min, m/z 74.00.

5. Data Processing

The Mass Hunter software was employed to extract peak areas and retention times. Standard curves were plotted, and the content of short-chain fatty acids in the samples was calculated.
